# Effects of After-School Basketball Program on Physical Fitness and Cardiometabolic Health in Prepubertal Boys

**DOI:** 10.3390/sports13090291

**Published:** 2025-08-28

**Authors:** Cristina Castro-Collado, Jose Manuel Jurado-Castro, Mercedes Gil-Campos, Belén Pastor-Villaescusa, Gracia María Quintana-Navarro, Francisco Jesús Llorente-Cantarero

**Affiliations:** 1 University of Cordoba, Maimonides Biomedical Research Institute of Cordoba (IMIBIC), Reina Sofia University Hospital, Avda Menéndez Pidal sn, 14004 Córdoba, Spain; colladocac@gmail.com (C.C.-C.); juradox@gmail.com (J.M.J.-C.); mbpastor.ugr@gmail.com (B.P.-V.); g.quintana.navarro@gmail.com (G.M.Q.-N.); fllorente@uco.es (F.J.L.-C.); 2 Consorcio CIBER, M.P. Fisiopatología de la Obesidad y Nutrición (CIBEROBN), Instituto de Salud Carlos III (ISCIII), 28029 Madrid, Spain; 3 Ciencias de la Actividad Física y El Deporte, Escuela Universitaria de Osuna (Centro Adscrito a la Universidad de Sevilla), 41640 Osuna, Spain; 4 Primary Care Interventions to Prevent Maternal and Child Chronic Diseases of Perinatal and Developmental Origin (RICORS), RD21/0012/0008, Instituto de Salud Carlos III (ISCIII), 28029 Madrid, Spain; 5 Spanish Network in Maternal, Neonatal, Child and Developmental Health Research (RICORS-SAMID, RD24/0013/0007), Instituto de Salud Carlos III (ISCIII), 28029 Madrid, Spain; 6 Department of Specific Didactics, Faculty of Education, University of Cordoba, 14071 Córdoba, Spain

**Keywords:** child, physical activity, body composition, exercise, cardiovascular diseases

## Abstract

Objectives: This study aimed to assess changes in anthropometric measures, cardiometabolic markers, and physical fitness following a structured basketball training program in healthy prepubertal boys. Methods: The intervention consisted of a 6-week pre-season phase followed by a 32-week basketball training season conducted during the academic year. Training sessions were held three times per week at moderate to vigorous intensity, along with a weekly match. The participants were assessed at baseline, 6, 9, and 12 months. A reference group was evaluated at baseline for comparison. The study was registered at ClinicalTrials.gov (identifier: NCT07007624). Results: Seventeen boys completed the program. Anthropometric assessments revealed increases in fat-free mass in the trunk and lower limbs, along with maintenance of an adequate BMI. After nine months, participants in the intervention showed significant improvements in fitness tests, including a 45% increase in Course Navette performance (*p* < 0.001), a 21% increase in horizontal jump performance (*p* = 0.001), and a 13% increase in abdominal test performance (*p* < 0.001). Conclusion: These findings suggest that a structured, school-based basketball program may enhance physical fitness and support healthy body composition maintenance in healthy-weight prepubertal boys.

## 1. Introduction

The participation of children in group or organized physical activities plays a fundamental role in their holistic development [1]. Beyond promoting physical health, participation in such activities also fosters emotional well-being, enhances social skills, and supports cognitive development by encouraging cooperation, discipline, and problem-solving abilities within a structured environment [2]. However, there is a concerning trend, as many children are not regularly involved in such activities. This lack of participation can be attributed to various factors, including the increasing dependence on technology and the limited access to suitable sports programs [3]. Understanding the causes and seeking solutions to encourage active participation of children in group physical activities are crucial for promoting a healthy lifestyle and an optimal development of social skills from an early age [4].

Regular physical activity improves physical fitness, including cardiovascular endurance, muscular strength, flexibility, lung capacity, body composition, and metabolic health [5,6]. Team sports are highly effective in improving youth health by enhancing strength, flexibility, balance, and coordination, while also reducing body fat and boosting cardiorespiratory endurance [7]. Basketball, in particular, is a comprehensive sport that involves sprinting, jumping, and feints, engaging both the upper and lower limbs [8,9]. As a team-based activity, it maintains active participation and high levels of physical activity [10]. Its high-intensity training and regular practice have been shown to produce significant improvements in physical fitness, even in children [11].

Intensive and frequent physical training can significantly influence cardiovascular health in children and adolescents [12]. While some studies focus exclusively on cardiovascular adaptations to training, there is limited research considering the effects of physical fitness on metabolism and body composition. Therefore, the objective of this study was to evaluate changes in physical fitness, cardiometabolic health, and body composition in boys participating in a regular after-school basketball training.

## 2. Materials and Methods

### 2.1. Study Design and Participants

A prospective, non-randomized controlled intervention study was conducted. The protocol was registered at ClinicalTrials.gov (NCT07007624). The study was designed and reported following the TREND statement guidelines (Supplementary Materials S1).

An after-school basketball program was offered to students from middle socioeconomic backgrounds in Córdoba (Spain). The inclusion criteria were age between 7 and 12 years, normal weight, and good health, with Tanner stage I (prepuberal). Exclusion criteria included any clinical or analytical sign of puberty, presence of disease, or use of medication that could affect blood pressure (BP), glucose, or lipid metabolism. The study was designed in accordance with the ethical principles for human research of the Declaration of Helsinki. It was approved by the local ethics and research committee at University Reina Sofia Hospital (Code: 1866). All participants and their families were informed about the study and voluntarily agreed to participate. Informed consent from their parents or legal guardians was obtained. All measurements and data collection were performed across the school year, from September to June.

A non-randomized convenience sample was selected due to ethical and logistical limitations that prevented the random assignment of children to a structured, year-long training program. First, 20 boys of the same age and gender were chosen to form a basketball team (group B), in accordance with federal recommendations. Their training sessions were monitored by a highly qualified basketball trainer. These boys were selected based on their interest in participating in basketball activities, which may have contributed to higher baseline levels of physical fitness. Three participants from the intervention group withdrew from the study due to scheduling conflicts. Their baseline anthropometric and physical fitness values were within the same range as the other interventions. Therefore, no relevant differences were identified that would suggest a systematic bias resulting from dropout. These participants were not included in the statistical analysis.

Second, a cohort of 74 boys from the same schools and social background was selected to serve as a healthy reference group (HRG), at baseline time. The purpose of including this group was to confirm that participants in the intervention group were comparable, at baseline, to a larger population of healthy prepubertal boys. All participants were healthy prepubertal boys of similar age and socioeconomic background. Although no formal matching was performed, both groups shared a common educational and social context. This healthy reference group (HRG) participated in a single assessment visit, as only one-time permission was granted.

### 2.2. Intervention

The basketball training program, supervised by a qualified trainer, was divided into two periods: a pre-season period at the beginning of the school year, during the first 6 weeks, and immediately afterward, a 32-week season lasting until the end of the school year. After the program concluded, recommendations to be active were provided for the summer holidays, and a follow-up assessment was conducted 3 months after the end of the intervention.

The pre-season included 5 training sessions (Monday to Friday) per week of 2 h each. This intervention was focused on dribbling, defending, and shooting skills. The season included 3 training sessions per week of 2 h each (after school) and a match during the weekend. Each session was divided into two periods separated by a break of 10 min: the first period focused on individual skills with moderate- to high-intensity (60–85% of heart rate (HR)) exercises, and the second period focused on game tactics and strategy.

### 2.3. Outcomes

**Figure 1:**
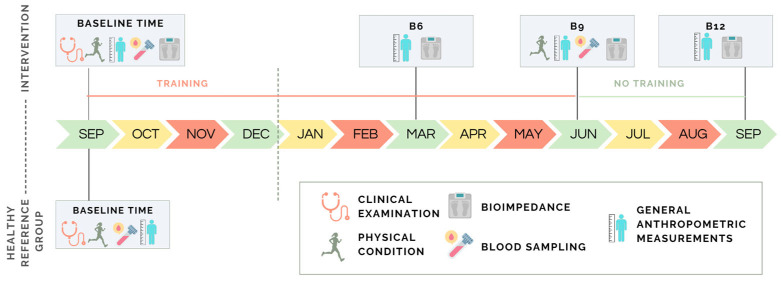
Activity schedule across the different time points of the study (basal B_0_, and at 6, 9, and 12 months (B_6_, B_9_, B_12_) in boys participating in a basketball program.

At baseline (B_0_), clinical examination, body composition, physical fitness tests, BP, and blood draws to assess biochemical parameters related to metabolism and cardiovascular health were performed in all boys included in the study. Follow-up visits were conducted at 6 months (B_6_), 9 months (B_9_), and 12 months (B_12_) during which anthropometric and other measurements were repeated. No training sessions took place between B_9_ and B_12_ (Figure 1). All measurements were performed under standardized conditions: after bladder emptying, 3 h after rising and eating, and without previous intensive exercise.

#### 2.3.1. Clinical Examination

A comprehensive clinical evaluation was performed by trained healthcare professionals, including a structured anamnesis to collect medical and family history (particularly regarding metabolic or cardiovascular diseases), current health status, and medication use. Sexual maturity was assessed using Tanner’s five-stage scale [13] through a physical examination conducted in a private setting by a pediatrician. In boys, this included the evaluation of genital development and pubic hair distribution.

#### 2.3.2. Body Composition

Body weight and height were measured using standard techniques, a beam balance, and a precision stadiometer SECA 213 (Scale 20–205 cm; SECA, Hamburg, Germany), with the participants lightly dressed and barefoot. BMI was calculated as weight (kg)/height (m^2^) and compared to growth charts for Spanish children; percentiles were calculated to determine the relative standing of each value within the data distribution [14]. The z-score was calculated to determine how far each value deviates from the mean in terms of standard deviations. Body composition components such as fat-free mass and fat mass were obtained by bioelectrical impedance analysis using a Tanita BC 418 MA Segmental Body Composition Analyser^®^ (TANITA^TM^, Tokyo, Japan).

#### 2.3.3. Blood Pressure

Systolic and diastolic BP and HR were measured in the right arm in a sitting position, using a random-zero sphygmomanometer (Dinamap V-100, GE HealthCare, Chicago, IL, USA) after the participants had rested without changing position for at least 5 min. Each measurement was taken three times non-consecutively. Total systolic blood pressure (SBP) and diastolic blood pressure (DBP) values were calculated as the average of all available data from both groups.

#### 2.3.4. Blood Biomarkers

Fasting blood samples were collected and analyzed for complete blood count, glucose, insulin, lipids, and the homeostatic model assessment for insulin resistance (HOMA-IR). Blood samples were obtained from the antecubital vein between 08.00 and 09.30 h after an overnight fast of at least 12 h. Samples collected in EDTA tubes were centrifuged and immediately stored at −80 °C. They were drawn. The blood count and general biochemical parameters were analyzed at the laboratory in the reference hospital. Glucose (coefficient of variation (CV) = 1.0%) was analyzed using the glucose oxidase method in an automatic analyzer (Roche-Hitachi Modular P and D Autoanalyser; Roche Laboratory Systems, Mannheim, Germany), and plasma insulin was analyzed by radioimmunoassay (RIA) (CV = 2.6%) using an automatic microparticle analyzer (AxSYM; Abbott Laboratories, Chicago, IL, US). Insulin resistance (IR) was calculated by the HOMA-IR. Serum triacylglycerides (TAG) (CV = 1.5%), total cholesterol (CV = 0.9%), high density lipoprotein cholesterol (HDL-c) (CV = 0.8%), and low-density lipoprotein cholesterol (LDL-c) (CV = 1.5%) were measured using an automatic analyzer (Roche-Hitachi Modular P and D Autoanalyser; Roche Laboratory Systems, Mannheim, Germany).

#### 2.3.5. Cardiorespiratory Fitness (Course Navette Test)

The Course Navette test was employed to evaluate cardiorespiratory fitness. Participants were required to run for the maximum possible time in a continuous 20 m shuttle run at a progressively increasing speed. The test is structured in periods or stages called *paliers*, each lasting approximately one minute, during which the running speed remains constant. At the end of each period, the speed increases slightly, requiring greater physical effort. The test ends when the participant fails to reach the line at the time of the beep on two consecutive occasions. The number of completed stages allows for an indirect estimation of maximal oxygen consumption (VO_2_ max) and enables the classification of aerobic performance into different levels [15].

#### 2.3.6. Lower Body Explosive Strength (Horizontal Jump Test)

Lower-body muscular strength is evaluated through the horizontal jump test [16]. Participants performed three maximal jumps with feet together; the longest was recorded. A take-off mark ensured correct foot placement and prevented invalid attempts.

#### 2.3.7. Core Strength (Abdominal Test)

Core strength was assessed using a 30 s sit-up test. From a supine position, participants performed as many correctly executed sit-ups as possible within the time limit, touching elbows to thighs and returning shoulders to the mat.

### 2.4. Sample Size

As the horizontal jump is one of the main outcomes for this study, the sample size was determined by calculating the statistical power based on a previous study [17], using G*Power software (version 3.1, Düsseldorf, Germany) for a repeated-measures ANOVA (within-subjects) design, with an expected effect size of f = 0.25, α = 0.05, power = 0.80 and four measurements. Based on this parameter, the minimum sample size required to detect an 8% difference in horizontal jump performance was estimated to be 17 participants. The horizontal jump was selected as the basis for the power calculation due to its high responsiveness to lower-body training and suitability for assessing neuromuscular adaptations in prepubertal children. Other parameters, such as metabolic markers, were considered exploratory and were not used for sample size determination because of their greater interindividual variability and lower sensitivity to exercise in a healthy pediatric population [18]. The aim was to assess the effectiveness of the intervention in improving physical fitness, which is why the horizontal jump test was chosen.

### 2.5. Statistical Analysis

Data are expressed as the mean ± standard deviation. The Shapiro–Wilk test was used to assess whether the variables followed a normal distribution. To compare physical fitness, anthropometric, and biochemical parameters at different time points within the intervention group, repeated-measures ANOVA was used for variables following a normal distribution, with Bonferroni-corrected post hoc tests applied when the main effect was significant. For variables that did not meet normality assumptions, the Wilcoxon test was used for within-group comparisons (e.g., B_0_ vs. B_12_). To compare baseline values between the HRG and the intervention group, the Mann–Whitney U test was applied, as the data did not follow a normal distribution and the sample sizes were unequal. Pearson correlation coefficients were employed to examine the bivariate correlations among the variables. Correlation models were fitted to assess relationships between physical fitness, anthropometry, and biochemical parameters at different times. All statistical analyses were performed using SPSS Statistics 25 software (IBM SPSS, New York, NY, US). In all analyses, statistical significance was set at a 2-tailed *p*-value < 0.05. A per-protocol analysis approach was used, and therefore, no imputation was applied for missing data. Only data from participants who completed the entire intervention, and all follow-up measurements were included in the analyses.

## 3. Results

**Figure 2:**
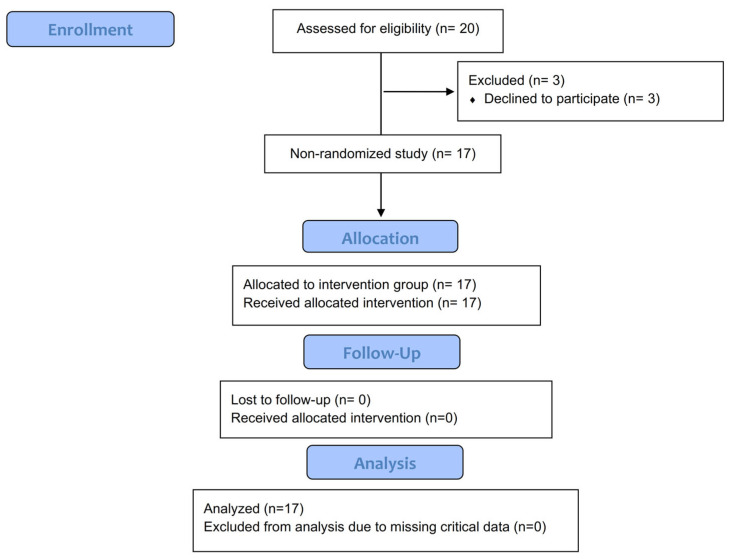
Flowchart of participants who completed the intervention.

A total of 91 children participated in the study: 74 in the HRG and 20 in the intervention group (B), of whom 17 completed the study (Figure 2). No significant differences observed between groups (HRG)—(B_0_) at baseline in weight, height, weight percentile, height percentile, BMI, and BMI z-scores. However, there was an increase in weight and height from B_0_ to B_12_ in trained boys. Additionally, lower levels in both SBP and DBP were noted in B_0_ compared to HRG and maintained during training (Table 1). The results of the HRG in comparison with B0 of the intervention group are presented in the Supplementary Materials (Supplementary Materials, Table S1).

**Figure 3:**
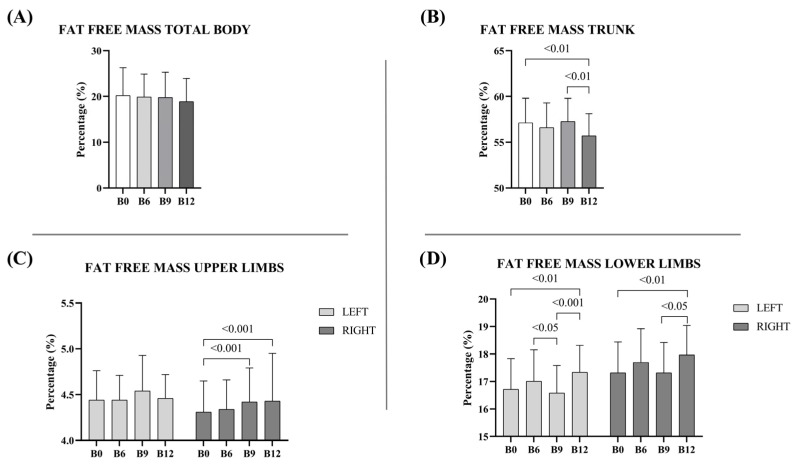
Percentage of fat-free mass at different times (B0, and at 6, 9, and 12 months (B6, B9, B12) in boys participating in the basketball training program. Data are expressed as mean percentages and standard deviation: (**A**) Fat-free mass total body; (**B**) Fat-free mass trunk; (**C**) Fat-free mass upper limbs; (**D**) Fat-free mass lower limbs. B0: baseline; B6: 6 months of intervention; B9: 9 months of intervention; B12: 3 months after the intervention (follow-up). Statistical significance was determined using one-way ANOVA or Wilcoxon test, depending on normality; *p*-values are reported for pairwise comparisons.

Significant differences in fat-free mass were observed in several body regions in boys trained at different times of measurements without differences in total fat-free mass (Figure 3A). Especially, in the trunk, a reduction in fat-free mass was found in B_12_ compared to B_9_ (B_9_: 57.26 ± 2.53% vs. B_12_: 55.71 ± 2.4%; Δ: 1.55%) and B_0_ (B_0_: 57.1 ± 2.71% vs. B_12_: 55.71 ± 2.4%; Δ: 1.39%) (Figure 3B). In upper limbs no significant differences were found except an increase in B_9_ (B_9_: 4.42 ± 0.37% vs. B_0_: 4.31 ± 0.34%; Δ: 0.11%) and B_12_ (B_12_: 4.43 ± 0.52% vs. B0: 4.31 ± 0.34%; Δ: 0.12%) respect to baseline in right limb (Figure 3C). Lower limbs showed an increase of fat-free mass from basal time to the end of the study (left (B_0_: 16.72 ± 1.11% vs. B_12_: 17.34 ± 0.97%; Δ: 0.62%); right (B_0_: 17.32 ± 1.12% vs. B_12_: 17.97 ± 1.07%; Δ: 0.65%)) (Figure 3D). Detailed data are presented in the Supplementary Materials (Supplementary Materials, Table S2).

**Figure 4:**
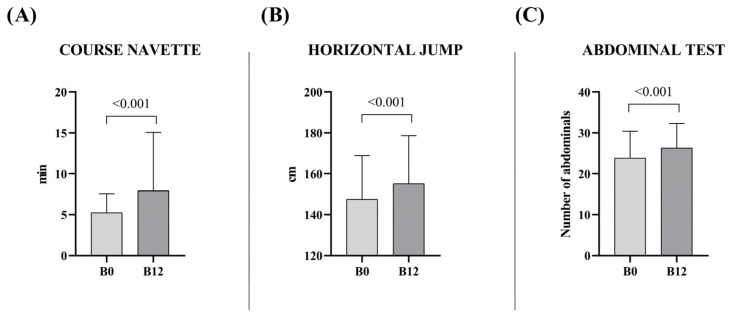
Physical fitness test results at baseline (B_0_) and after 12 months (B_12_) in boys participating in the basketball training program. Data are expressed as mean and standard deviation: (**A**) Course Navette; (**B**) Horizontal jump; (**C**) Abdominal test. Statistical significance was determined using one-way ANOVA or Wilcoxon test, depending on normality; *p*-values are reported for pairwise comparisons.

The group of basketball training showed better results in jump and abdominal tests than HRG at basal time without differences in Course Navette test (Supplementary Materials, Table S1). At B_12_, boys improved their results in all these tests compared with their baseline data (Course Navette (B_0_: 5.2 ± 2.3 min vs. B_12_: 7.9 ± 2.2 min; Δ: 2.7 min); horizontal jump (B_0_: 147.5 ± 21.3 cm vs. B_12_: 155.2 ± 23.5 cm; Δ: 7.7 cm); abdominal test (B_0_: 23.8 ± 6.6 number of repetitions vs. B_12_: 26.3 ± 6 number of repetitions; Δ: 2.5 number of repetitions) (Figure 4). The data are presented in detail in the Supplementary Materials (Supplementary Materials, Table S3).

Blood parameters were within the normal range compared with reference values from the laboratory, indicating that all the children exhibited a state of good health at baseline (for both groups). It was maintained in the follow-up after the intervention (B_12_) in the trained group (Table 2).

The Course Navette test was negatively correlated with BMI z-score at baseline (r = −0.521, *p* = 0.046), and this association became stronger at B_12_ (r = −0.738, *p* = 0.010), indicating that higher aerobic fitness was linked to lower BMI z-scores. At B_12_ follow-up, the Course Navette test also showed a significant inverse correlation with C-reactive protein (CRP) levels (r = −0.714, *p* = 0.009). In addition, at B_12_ follow-up_,_ both horizontal jump performance (r = −0.651, *p* = 0.022) and abdominal test performance (r = −0.659, *p* = 0.020) were negatively correlated with CRP.

## 4. Discussion

An after-school program of basketball training conducted during the school year led to improvements in body composition and physical fitness in a group of prepubertal boys, compared with a similar group that did not receive the intervention.

While few studies have explored anthropometric and performance adaptations specifically in preadolescent basketball players, most have involved shorter interventions, heterogeneous samples, or lacked follow-up assessments. In contrast, the present study adds novel evidence from a 9-month structured basketball program in healthy-weight prepubertal boys, including data from a post-intervention training period. This approach provides a clearer understanding of both the benefits and the potential reversibility of such interventions during a critical stage of development. Some limitations in evaluating the effects of basketball training are associated with small teams with a controlled number of participants [19] or teams solely comprised of individuals of one sex [20]. However, in this study, comparison with an HRG at baseline ensured that all participants began from similar conditions with comparable health statuses prior to training. Furthermore, the intervention was conducted by expert trainers who specifically prepared this group of prepubertal boys to compete under real conditions against similar counterparts.

Previous studies have consistently demonstrated a general increase in all anthropometric traits during the training period in children who practice basketball, coupled with a decrease in fat mass [21,22,23]. This reduction in fat mass has been attributed to both the rapid growth of fat-free mass and a slower accumulation of fat. Excessive fat mass not only hampers mobility in sports but also adversely affects physical performance [24]. Decreasing body fat reduces strain on both the musculoskeletal and cardiovascular systems, thereby enhancing movement efficiency and the capacity for physical exercise, which leads to improved strength, endurance, and overall functional capability [25]. In the children participating in this study, a decrease in BMI z-scores and fat mass was observed, while normal-weight status was maintained. These results were accompanied by improved performance in the Course Navette test. These findings are consistent with those of a previous study in children of similar age, where participants with higher BMI achieved worse results in fitness tests [26].

Basketball emerges as an excellent candidate for enhancing body composition and bone mineral density due to its high-impact nature [27]. Sports like basketball are characterized by their impact and required actions, such as jumping, similar to volleyball [28], handball [29], or dance [30]. Furthermore, full-body sports such as swimming involve both the upper and lower limbs but lack impact on the body, as they take place in a microgravity environment where the body is not subjected to impact forces [31]. In this study, a general improvement in fat-free mass was observed in both the lower and upper limbs throughout the training period. Participation in comprehensive sports that involve both lower and upper body training has been associated with a decrease in fat mass and an improvement in fitness levels among prepubertal and early pubescent children [32].

The Course Navette test is a widely used tool to assess physical fitness, providing valuable information on maximal effort and aerobic capacity [33]. It also reflects cardiovascular and respiratory health through its estimation of maximal oxygen consumption [34]. This improvement is attributed to their commitment to an active lifestyle, characterized by regular levels of physical activity. The systematic practice of sports also promotes cardiovascular health, which is further associated with anthropometric improvements [35]. In the present study, interesting inverse correlations have been found, especially at the end of the intervention, between physical fitness and both weight and BMI z-score, and with CRP, a well-established biomarker of inflammation. These findings suggest that improvements in physical fitness may be associated with healthier body composition and reduced systemic inflammation in children by the end of the intervention.

The frequency of sport practice has been closely linked to improvements in fitness [36]. A study involving 19 basketball players in training sessions conducted twice a week for 8 weeks resulted in enhanced cardiorespiratory fitness, explosive strength, and core strength [37]. Similarly, in another study focusing on prepubertal children who trained three times a week for 12 weeks, improvements were observed in anaerobic capacity, endurance running, and overall strength [32]. Thus, training twice a week emerges as a viable option to achieve health benefits with enhancements in jumping and running performances, along with an increase in fat-free mass, as previously reported [26]. Furthermore, higher performance in horizontal jumps has been linked to improved sprints and agility in male adolescent basketball players [38].

It is also evident that participants in the intervention group demonstrated superior performance compared to those in the HRG assessed at baseline, particularly in the abdominal and horizontal jump tests. This discrepancy may be attributed to the fact that individuals in the intervention group were generally more active and inclined to participate in sports activities compared to their HRG counterparts. This suggests a potential selection bias, as children with a greater inclination toward physical activity may have been more likely to enroll in the intervention, thereby limiting the attribution of the observed improvements solely to the training program.

Both groups showed normal biochemical values at baseline, which were maintained after the intervention in the basketball group. Regular, controlled training appears to support the maintenance of a healthy metabolic profile in children. Throughout the follow-up, no significant longitudinal changes were observed in the intervention group. Only CRP showed an inverse relationship with physical fitness outcomes, suggesting a potential link between inflammatory status and fitness. In particular, basketball benefits multiple systems and supports disease prevention during youth [39]. However, the primary health-related improvements observed in this intervention were related to physical fitness and body composition, rather than metabolic markers. These benefits seem to decline after ~12 weeks of inactivity, with reductions in lean mass evident within three months after the intervention (B_12_). Concretely, some outcomes, such as trunk fat-free mass, declined during the three-month follow-up after the intervention ended (B_12_), indicating that the positive effects of the program may be partially reversible. This regional decline may reflect the nature of basketball, which emphasizes lower-limb activity. Therefore, reductions in trunk fat-free mass after detraining may have occurred more rapidly compared to the legs. These findings highlight the importance of maintaining regular physical activity to sustain the benefits achieved through structured training interventions. Furthermore, future studies should continue to explore whether dynamic and high-intensity sports such as basketball might positively influence metabolic health and insulin sensitivity [40].

### 4.1. Practical Implications

Maintaining regular physical activity is essential for preventing sedentary behaviors that increase the risk of chronic diseases. This study supports basketball as an effective strategy to promote physical fitness and maintain healthy body composition in healthy-weight prepubertal children. These findings could help inform recommendations in pediatric healthcare settings, particularly for children at risk of excess adiposity or low fitness levels. Future public policies might consider incorporating structured after-school sports programs as part of preventive health strategies for youth populations.

The program was implemented by certified basketball coaches with extensive experience in youth sports. Rather than limiting replicability, we believe this detail strengthens the internal validity of the study and sets a benchmark for structured school-based interventions. This characteristic may serve as a model for future programs aiming to replicate similar outcomes under comparable conditions.

### 4.2. Limitations

This study was limited to prepubertal boys, which may reduce the generalizability of findings to girls or older adolescents. The intervention was conducted in a single setting with a relatively small sample size. Participation was voluntary, and children with greater initial motivation or physical fitness may have been more likely to participate (e.g., interest in participating in basketball activities), introducing a selection bias that could explain baseline differences in some physical fitness variables. Moreover, due to ethical and logistical constraints, it was not feasible to randomly assign children to an extracurricular training program they might not have chosen to join, which limited the possibility of randomization. Future studies should include longitudinal follow-ups to assess sustained effects and explore differences by sex, maturation stage, and training modalities. Another important limitation is the absence of a longitudinal control group. The HRG was included at baseline only to ensure a similar health status to the group that would participate in the program, but it was not followed over time. Therefore, we cannot completely rule out the influence of natural growth and development on the observed changes in the intervention group. Heart rate monitoring was not conducted because it is prohibited in basketball for safety reasons, both during competitions and training sessions, as it is a high-contact sport. Additionally, in this type of sport, it is difficult to accurately assess the intensity at which each participant is exercising. However, by using physical fitness tests to assess the participants’ baseline capacity, the training intensity and volume were adjusted to approximately 60–85%, based on their age. This approach ensures that participants remain within an aerobic range, avoiding excessive intensity that could push them into an anaerobic zone. All sessions were supervised by a qualified instructor with a degree in sport sciences. However, all sessions were supervised by a certified basketball coach with a degree in sport sciences. Additionally, the study included only a single follow-up measurement after the intervention ended (B_12_), which limited the ability to evaluate the long-term sustainability of the observed benefits.

## 5. Conclusions

A 32-week after-school basketball training program conducted three times per week, plus a weekend match, and performed at moderate to vigorous intensity, was associated with improvements in physical fitness and body composition in healthy-weight prepubertal boys. However, randomized controlled interventions should be conducted, and meanwhile, these changes should be interpreted with caution, as natural growth and other external factors may have contributed to the observed effects. Nevertheless, these results support the integration of structured team sports like basketball into school or community programs as an effective strategy to promote children’s health and prevent the early onset of metabolic disorders.

## Figures and Tables

**Table 1:** General anthropometric measurements at baseline (B_0_) and at 12-month follow-up (B_12_) in the intervention group after the basketball training program.

	B_0_	B_12_	Δ	*p*-Value
Age (year)	10.9 ± 1.41	11.9 ± 1.41	1.0	<0.001
Weight (kg)	43.8 ± 10.45	47.0 ± 10.80	3.2	<0.001
Weight percentile	56.8 ± 28.32	54.7 ± 28.61	2.1	0.826
Height (cm)	149.4 ± 8.67	153 ± 9.83	3.6	<0.001
Height percentile	65.2 ± 26.09	68.0 ± 9.83	2.8	0.361
BMI (kg/m^2^)	19.5 ± 3.04	19 ± 2.57	0.5	0.555
BMI Z-score	0.1 ± 0.96	−0.1 ± 0.73	0.2	0.099
DBP (mmHg)	62.9 ± 8.90	55 ± 5.99	2.1	0.311
SBP (mmHg)	114.5 ± 7.68	108.2 ± 10.63	6.3	0.450
HR (bpm)	66.4 ± 11.57	69.7 ± 10.84	3.3	0.005

BMI: Body mass index; DBP: Diastolic blood pressure; SBP: Systolic blood pressure; HR: Heart rate. Data are expressed as mean ± standard deviation and differences (∆: B_12_–B_0_); One-way ANOVA or Wilcoxon test was performed, depending on whether the variables followed a normal distribution, using repeated measures.

**Table 2:** Blood parameters at baseline (B_0_) and at 12-month follow-up (B_12_) in the intervention group after the basketball training program.

	B_0_	B_12_	Δ	*p*-Value	Reference Values
Leukocytes (×10^3^/µL)	6.13 ± 1.56	5.49 ± 1.34	0.64	0.055	4–12.4
Hemoglobin (g/dL)	12.82 ± 2.37	13.11 ± 0.75	0.29	0.226	12–16
Iron (mg/dL)	60.50 ± 26.70	78.82 ± 21.17	18.32	0.016	50–170
Ferritin (ng/mL)	30.78 ± 12.98	30.24 ± 13.36	0.51	0.776	10–120
Glucose (mg/dL)	90.15 ± 14.46	92.29 ± 7.08	2.14	0.736	74–106
Insulin (µUI/mL)	8.09 ± 6.15	8.90 ± 3.59	0.81	0.962	3–25
HOMA-IR	1.89 ± 1.34	2.04 ± 0.86	0.15	0.981	0–2.9
Total cholesterol (mg/dL)	169.40 ± 27.83	166.18 ± 26.61	3.22	0.434	140–220
HDL-c (mg/dL)	64.15 ± 11.18	52.82 ± 11.49	11.33	<0.001	40–80
LDL-c (mg/dL)	91.75 ± 24.11	99.18 ± 23.42	7.43	0.077	50–130
Apolipoprotein a (mg/dL)	148.26 ± 23.07	129.88 ± 19.31	18.38	<0.001	105–220
Apolipoprotein b (mg/dL)	60.37 ± 14.66	61.06 ± 12.92	0.69	0.966	65–130
Triglycerides (mg/dL)	65.25 ± 20.30	68.88 ± 31.00	3.63	0.467	20–250
Aspartate Transaminase (U/L)	26.10 ± 5.31	22.82 ± 3.15	3.28	0.003	5–34
Alanine transaminase (U/L)	20.20 ± 11.15	17.35 ± 5.58	2.85	0.115	10–49
C-reactive protein (mg/dL)	2.55 ± 4.35	0.92 ± 1.25	1.63	0.017	0–10

HOMA-IR: Homeostasis model assessment of insulin resistance; HDL-c: High density lipoprotein cholesterol; LDL-c: Low density lipoprotein cholesterol. Data are expressed as mean ± standard deviation and differences (∆: B_12_–B_0_); one-way ANOVA or Wilcoxon test was performed, depending on whether the variables followed a normal distribution, using repeated measures.

## Data Availability

The data presented in this study are available on request from the corresponding author. The data are not publicly available due to the patient privacy policy.

## Supplementary Materialss

The following supporting information can be downloaded at: https://www.mdpi.com/article/10.3390/sports13090291/s1, Table S1: Descriptive characteristics of the reference group; Table S2: Percentage of fat free mass in different times (basal B0, and at 6, 9 and 12 months (B6, B9, B12) in boys trained in a basketball program; Table S3: Physical fitness tests data at basal time in the intervention group (B0), and after a year of follow-up in the boys trained in the basketball program (B12).

## Abbreviations

The following abbreviations are used in this manuscript:

BMI	Body mass index
DBP	Diastolic blood pressure
SBP	Systolic blood pressure
HR	Heart rate
HOMA-IR	Homeostasis model assessment of insulin resistance
HDL-c	High density lipoprotein cholesterol
LDL-c	Low density lipoprotein cholesterol
