# Supplementary material for: Effects of After-School Basketball Program on Physical Fitness and Cardiometabolic Health in Prepubertal Boys
Source: Sports (Basel). 2025 Aug 28;13(9):291. doi: 10.3390/sports13090291 (PMC12473969; doi:10.3390/sports13090291)
Supplement: Supplementary file 1 [file sports-13-00291-s001.zip › sports-3787503-supplementary.pdf]

Supplementary Material S1

Table S1: Descriptive characteristics of the reference group.

	**HRG**	**B0**	**Δ**	***p*-value**
**Age (year)**	10.7 ± 0.94	10.49 ± 0.71	0.21	**0.019**
**Weight (kg)**	45.7 ± 12.77	43.8 ± 10.45	1.90	0.656
**Weight percentile**	63.3 ± 31.85	56.8 ± 28.32	6.50	0.303
**Height (cm)**	147.0 ± 9.80	149.4 ± 8.67	2.40	0.155
**Height percentile**	59.8 ± 35.64	65.2 ± 26.09	5.40	0.898
**BMI (kg/m²)**	20.8 ± 3.76	19.5 ± 3.04	1.30	0.086
**BMI Z-Score**	0.8 ± 1.23	0.56 ± 0.95	0.24	0.236
**DBP (mmHg)**	69.3 ± 9.17	62.9 ± 8.90	6.40	**0.008**
**SBP (mmHg)**	126.3 ± 13.14	114.5 ± 7.68	11.8	**<0.001**
**HR (bpm)**	82.0 ± 13.24	66.4 ± 11.57	15.6	**<0.001**
**BLOOD PARAMETERS**				
**Leukocytes (x10^3/µL)**	6.49 ± 1.67	6.13 ± 1.56	0.36	0.408
**Hemoglobin (g/dL)**	13.34 ± 1.39	12.82 ± 2.37	0.52	0.152
**Iron (mg/dL)**	77.05 ± 30.14	60.50 ± 26.70	16.55	**0.006**
**Ferritin (ng/mL)**	31.58 ± 15.61	30.78 ± 12.98	0.80	0.838
**Glucose (mg/dL)**	87.21 ± 12.79	90.15 ± 14.46	2.94	**0.036**
**Insulin (µUI/mL)**	7.24 ± 5.11	8.09 ± 6.15	0.85	0.510
**HOMA-IR**	1.49 ± 1.12	1.89 ± 1.34	0.40	0.070
**Total cholesterol (mg/dL)**	168.87 ± 22.07	169.40 ± 27.83	0.53	0.906
**HDL-c (mg/dL)**	57.10 ± 11.15	64.15 ± 11.18	7.05	**0.001**
**LDL-c (mg/dL)**	91.75 ± 24.11	91.75 ± 24.11		0.080
**Apolipoprotein a (mg/dL)**	145.98 ± 19.17	148.26 ± 23.07	2.28	0.615
**Apolipoprotein b (mg/dL)**	70.60 ± 12.90	60.37 ± 14.66	10.23	**0.001**

**Triglycerides (mg/dL)**	64.38 ± 25.28	65.25 ± 20.30	0.87	0.440
**Aspartate Transaminase (U/L)**	26.73 ± 5.14	26.10 ± 5.31	0.63	0.542
**Alanine transaminase (U/L)**	19.44 ± 16.27	20.20 ± 11.15	0.76	0.475
**C-reactive protein (mg/dL)**	1.88 ± 3.29	2.55 ± 4.35	0.67	0.050
**PHYSICAL CONDITION**				
**Course Navette (min)**	5..04 ± 2.33	5..24 ± 2.31	0.20	0.781
**Horizontal Jump (cm)**	128..78 ± 18.85	147..50 ± 21.30	18.72	**0.002**
**Abdominal Test (Number of abdominals)**	15..27 ± 5.214	23..83 ± 6.60	8.56	**<0.001**

BMI: Body Mass Index; DBP: Diastolic blood pressure; SBP: Systolic blood pressure; HR: Heart rate; HOMA-IR: Homeostasis Model Assessment of Insulin Resistance; HDL-c: High density lipoprotein cholesterol; LDL-c: Low density lipoprotein cholesterol. Data are expressed as mean ± standard deviation. The Mann-Whitney U test was used to calculate the p-values.

Table S2: Percentage of fat free mass in different times (basal B_0, and at 6. 9 and 12 months (B_6, B_9, B_{12}) in boys trained in a basketball program.

	**B0**	**B6**	**B9**	**B12**	***p*-value**
**FFM total (%)**	79.83 ± 6.09	80.13 ± 5.01	80.28 ± 5.52	81.13 ± 5.04	0.324
**FFM RUL (%)**	4.31 ± 0.34	4.34 ± 0.32	4.42 ± 0.37	4.43 ± 0.52	**0.038**
**FFM LUL (%)**	4.44 ± 0.32	4.44 ± 0.27	4.54 ± 0.39	4.46 ± 0.26	0.214
**FFM LLL (%)**	16.72 ± 1.11	17.01 ± 1.14	16.58 ± 1.00	17.34 ± 0.97	**<0.001**
**FFM RLL (%)**	17.32 ± 1.12	17.69 ± 1.23	17.32 ± 1.10	17.97 ± 1.07	**0.010**
**FFM Trunk (%)**	57.10 ± 2.71	56.59 ± 2.70	57.26 ± 2.53	55.71 ± 2.40	**0.006**

FFM: Fat-Free Mass; RUL: Right Upper Limbs; RLL: Right Lower Limbs; LUL: Left Upper Limbs; LLL: Left Lower Limbs. Data are expressed as mean ± standard deviation. The Mann-Whitney U test was used to calculate the p-values.

Table S3: Physical fitness tests data at basal time in the intervention group (B_0), and after a year of follow-up in the boys trained in the basketball program (B_{12}).

	T1	T2	*p*-value
**Course Navette (min)**	5.24 ± 2.31	7.92 ± 2.19	**<0.001**
**Horizontal Jump (cm)**	147.50 ± 21.30	155.17 ± 23.49	**<0.001**
**Abdominal Test ((Number of abdominals)**	23.83 ± 6.60	26.33 ± 5.97	**<0.001**

Data are expressed as mean ± standard deviation. The Mann-Whitney U test was used to calculate the p-values.
